# Supplementary material for: Permissive zones for the centromere-binding protein ParB on the Caulobacter crescentus chromosome
Source: Nucleic Acids Res. 2017 Nov 23;46(3):1196–209. doi: 10.1093/nar/gkx1192 (PMC5815017; doi:10.1093/nar/gkx1192)
Supplement: Supplementary Data [file gkx1192_supp.pdf]

Permissive zones for the centromere-binding protein ParB on the *Caulobacter crescentus* chromosome

Ngat T. Tran[1‡], Clare E. Stevenson[2‡], Nicolle F. Som[1], Anyarat Thanapipatsiri[1], Adam S. B. Jalal[1], and Tung B. K. Le[1*]

[1]Department of Molecular Microbiology

[2]Department of Biological Chemistry

John Innes Centre, Norwich, NR4 7UH, United Kingdom

[‡]Co-first authors

[*]Correspondence: tung.le@jic.ac.uk

SUPPLEMENTARY FIGURES AND LEGENDS

SUPPLEMENTARY MATERIALS AND METHODS

SUPPLEMENTARY TABLES S1-S3

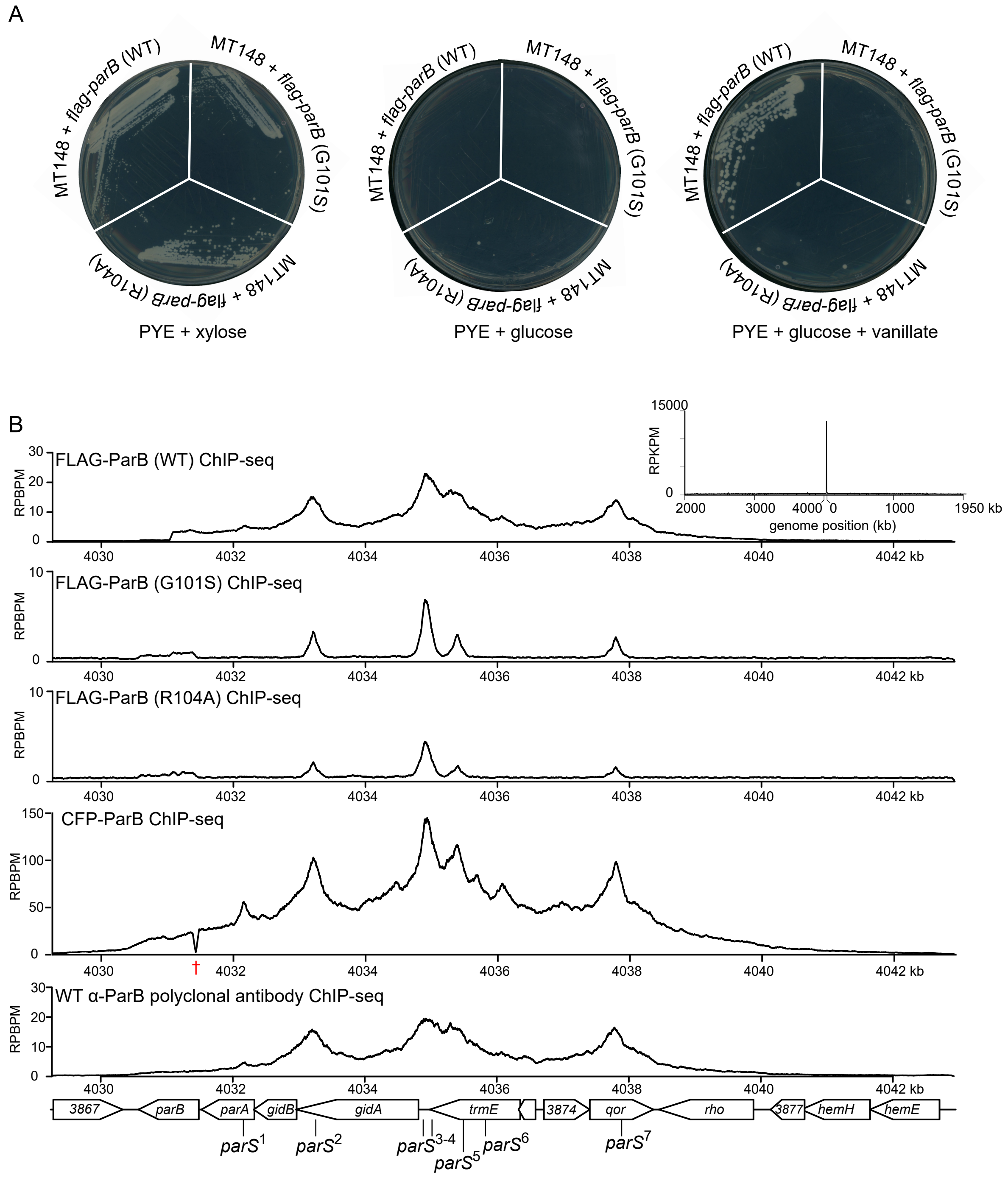

Figure S1. Genomic distributions of wild-type *Caulobacter* ParB and spreading-defective ParB (G101S) and ParB (R104A) variants. (A) *Caulobacter* strains *parB::*P_{xyl}*-parB van::*P_{van}*-flag-parB* WT, G101S, or R104A were restruck on PYE + xylose to induce the expression of the wild-type untagged ParB, or on PYE + glucose to repress the expression of the wild-type untagged ParB, or on PYE + glucose + vanillate to repress the expression of the wild-type untagged ParB while expressing the FLAG-tagged ParB WT, G101S, or R104A. The FLAG-tagged version of wild-type ParB is functional and can complement the depletion of wild-type untagged ParB while the spreading mutant ParB (G101S) or ParB (R1014A) cannot. **(B)** ChIP-seq profiles of FLAG-ParB (WT), FLAG-ParB (G101S), and (R104A) (using α-FLAG antibody), of CFP-ParB (using α-GFP antibody), and of ParB (using polyclonal α-ParB antibody). Note: the red dagger (†) symbol on the CFP-ParB ChIP-seq profile indicates the genomic region where sequencing reads were missing. This is because CFP-ParB ChIP-seq reads were mapped to the wild-type *Caulobacter* reference genome instead of to the genome of *parB::cfp-parB* strain.

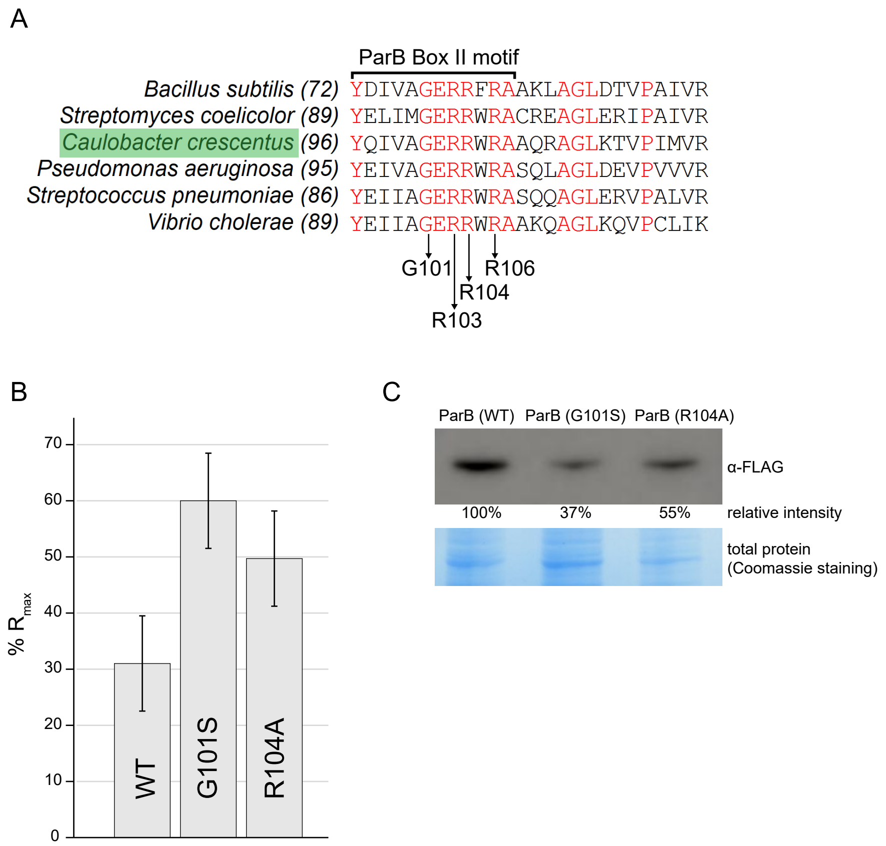

Figure S2. Identification of potential spreading-defective ParB mutants in *Caulobacter crescentus*. (A) A sequence alignment of *Caulobacter* ParB and homologs to highlight the conservation of the Box II motif (square bracket). Identical amino acids residues are shown in red. Vertical arrows indicate the position of G101, R103, R104, and R106 residues. **(B)** Surface Plasmon Resonance (SPR) was used to measure binding affinity of ParB WT, ParB (G101S) and ParB (R104A) at 200 nM to a 24-bp double-stranded DNA that contains *parS* site 4. The level of ParB variants binding to DNA was expressed as a percentage of the theoretical maximum response, R_{max}, assuming a single ParB dimer binding to one immobilized double-stranded DNA oligonucleotides. This normalization process enabled the various responses to be readily compared, irrespective of the quantity and length of the DNA tethered on an SPR chip surface. **(C)** Immunoblot analysis of FLAG-tagged ParB WT vs. G101S and R104A. Cells were depleted of wild-type untagged ParB for 5 hours, then vanillate was added for an additional hour to allow for expression of FLAG-tagged ParB. Equal amount of total protein was loaded on each well of the SDS-PAGE. The relative immunoblot intensity is indicated below each band.

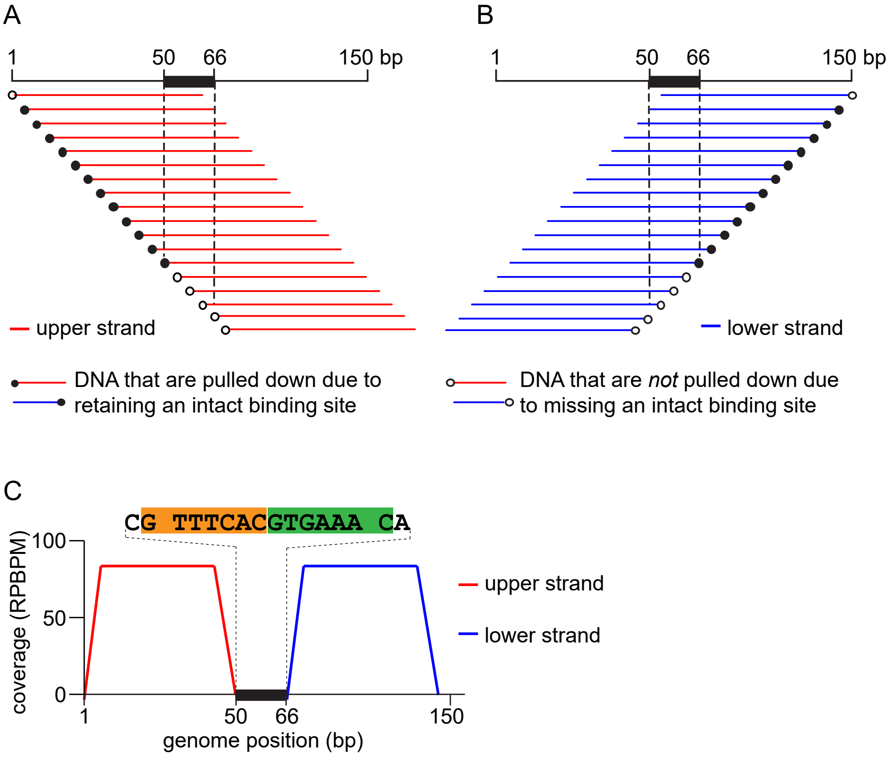

Figure S3. Methodology for the analysis of *in vitro* affinity purification with deep sequencing (IDAP-seq) data. Sequencing reads were sorted to either the upper DNA strand **(A)** or to the lower strand **(B)** of the *Caulobacter* reference genome (6). The 5' nucleotides are shown as circles. Solid circles are for DNA fragments with an intact ParB binding site (black rectangle), and open circles are for DNA fragments with a partial or no ParB binding site. Only DNA fragments with an intact ParB binding site will be pulled down during affinity purification and contribute to the sequencing coverage. **(C)** A schematic strand-specific coverage map of IDAP-seq. A footprint of ParB can be identified in between the two edges of the upper-strand peak (red) and the lower-strand peak (blue). The schematic picture was adapted from Belitsky and Sonenshein (2013) (31) with permission from A. Sonenshein.

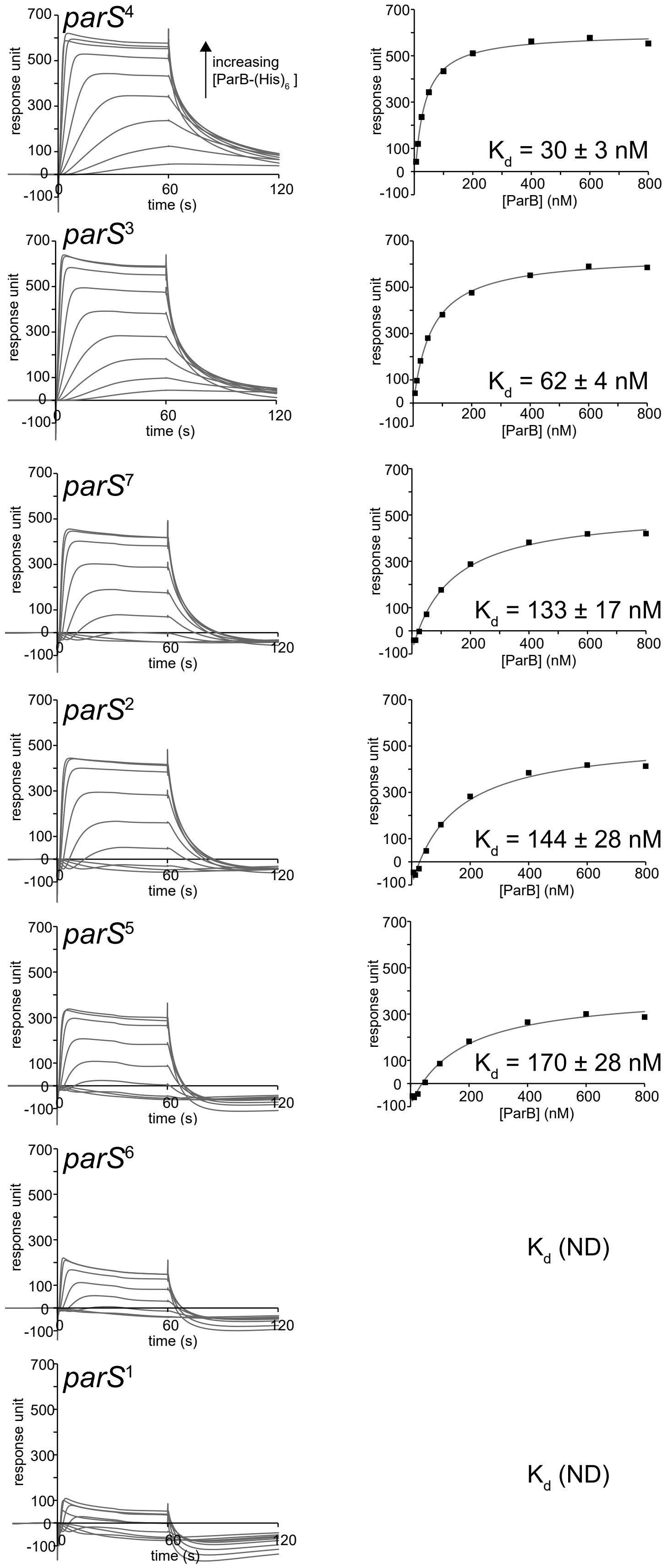

Figure S4. Determination of binding affinity constants (K_d) of *Caulobacter crescentus* ParB-*parS* interactions. 24-bp duplex DNA containing each individual *parS* was tethered on an SPR chip surface. Increasing concentrations of ParB (6.25 nM, 12.5 nM, 25 nM, 50 nM, 100 nM, 200 nM, 400 nM, 600 nM and 800 nM) were flown through the SPR chip surface. Binding of ParB to *parS* site was recorded and expressed as response unit (RU). Response units were plotted against ParB concentration and curve fitted to estimate K_d value ± standard deviation. K_d for *parS* site 1, site 6, and the scrambled *parS* site 3 were not determined due to very little specific binding of ParB to the tested DNA.

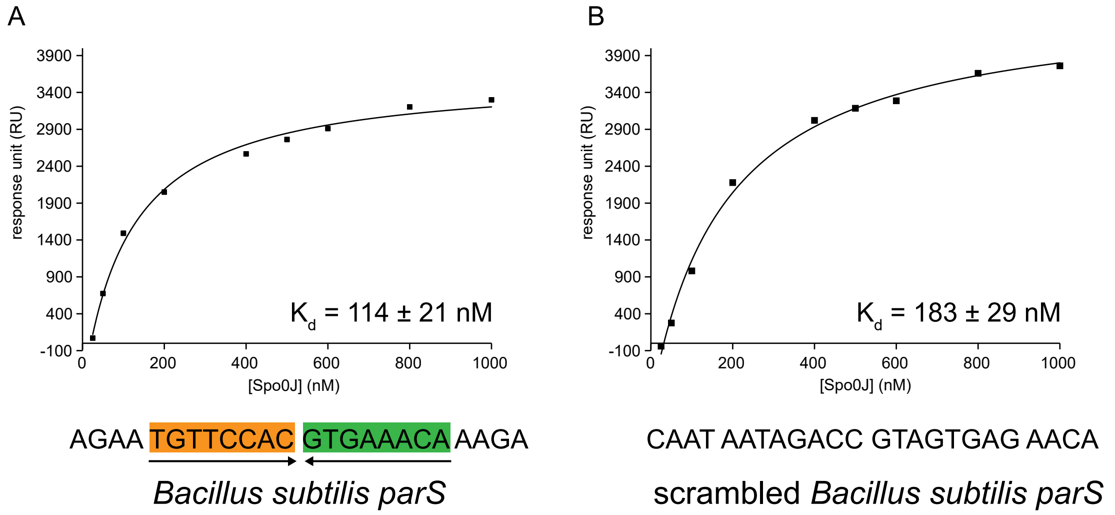

Figure S5. Determination of binding affinity constants (K_d) of *Bacillus subtilis* Spo0J/ParB-*parS* interactions. 24-bp duplex DNA containing either **(A)** a *B. subtilis parS* or **(B)** a scrambled *parS* site were tethered on an SPR chip surface. Increasing concentrations of *B. subtilis* Spo0J (25 nM, 50 nM, 100 nM, 200 nM, 400 nM, 500 nM, 600 nM, 800 nM, and 1000 nM) were flown through the SPR chip surface. Binding of Spo0J to *parS* site was recorded and expressed as response unit (RU). Response units were plotted against Spo0J concentration and curve fitted to estimate the K_d value ± standard deviation. The purified Spo0J that has the same linker peptide and 6xHis at the C-terminus as the purified *Caulobacter* ParB. The *B. subtilis parS* chosen for this study is at 359° (at 4,205,736 bp on the *B. subtilis* 168 genome). The core 16-bp *parS* site of *parS* 359° is identical to the strongest *Caulobacter parS* site 4, enabling a direct comparison between ParB from the two species.

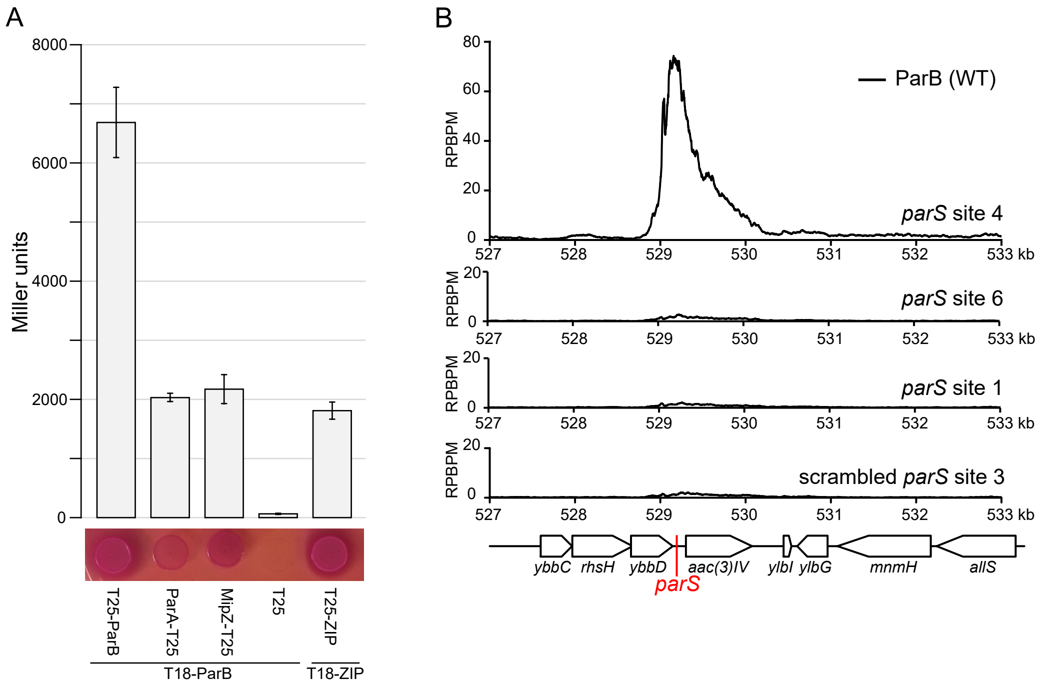

Figure S6. T18-ParB is functional in *Escherichia coli.* (A) A ParB protein was expressed from an IPTG-inducible promoter as a C-terminal fusion to the T18 fragment of *Bordetella pertussis* adenylate cyclase. Known interacting partners of ParB were expressed as fusion proteins to the T25 fragment of *B. pertussis*: T25-ParB, ParA-T25 and MipZ-T25. Interactions between ParB and partners were assessed on a solid MacConkey agar or by β-galactosidase assay. Three biological replicates were performed for each pair of interacting partners. A negative control (T25 fragment alone) and a positive control: T25-ZIP and T18-ZIP were also included. **(B)** ChIP-seq profiles of T18-ParB at *parS* site 4, site 6 and site 1 in an *E. coli* heterologous host. T18-ParB protein was expressed by addition of 500 μM IPTG for an hour before fixing with formadehyde for ChIP-seq. DNA bounds to T18-ParB was immunoprecipitated using α-T18 conjugated sepharose beads. A scrambled *parS* site 3 was also inserted at the *ybbD* locus to serve as a negative control. ChIP-seq signals were reported as the number of reads at every nucleotide along the genome (RPBPM value).

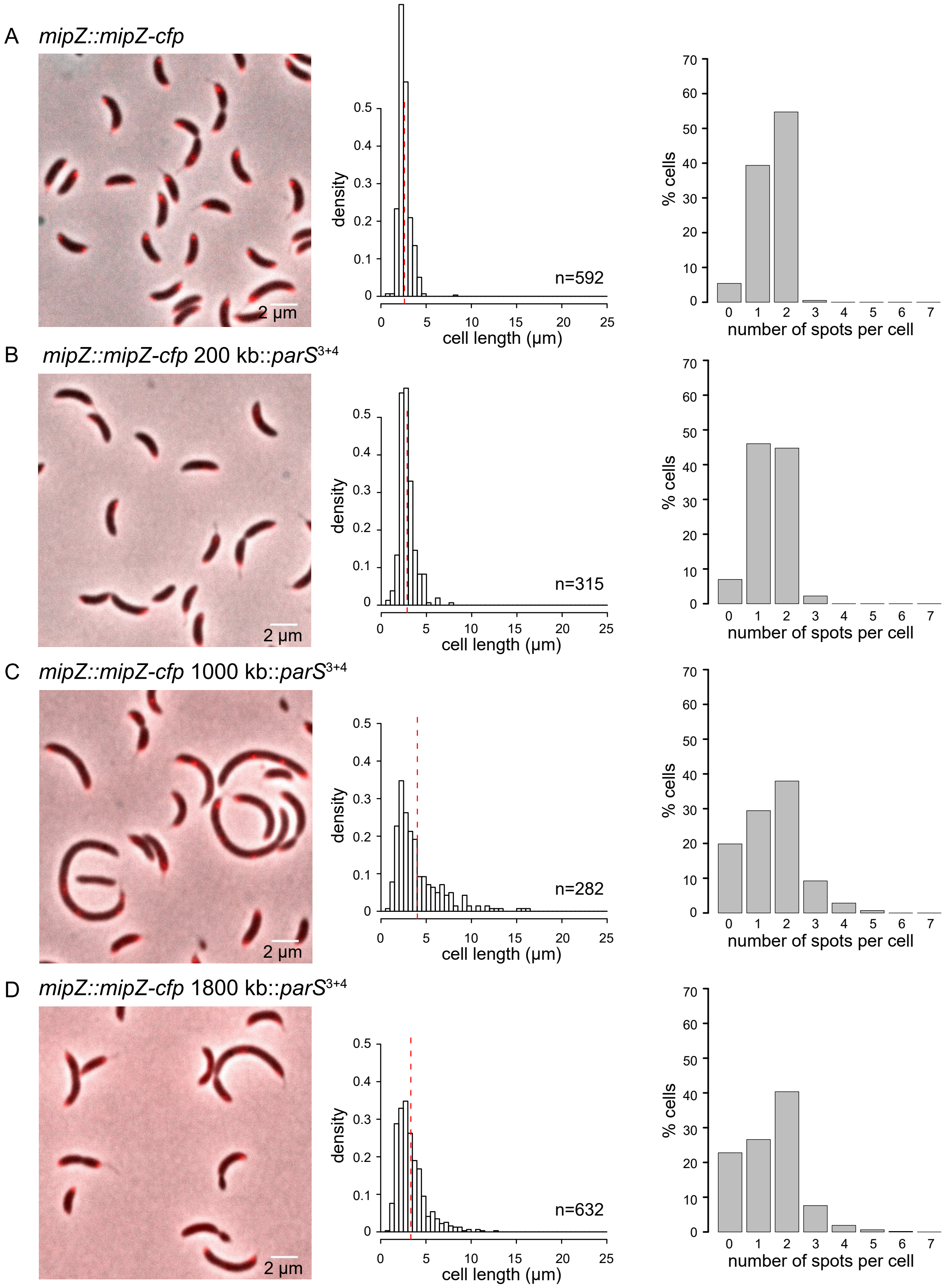

Figure S7. The position of an ectopic *parS* on the chromosome is critical for the fitness of *Caulobacter*

Micrograph of *mipZ::mipZ-cfp Caulobacter* cells **(A)** without an extra ectopic *parS*$^{3+4}$, **(B)** with an extra ectopic *parS*$^{3+4}$ at +200 kb, **(C)** at +1000 kb, or **(D)** at +1800 kb. Cell length of an exponentially-growing cells were quantified and presented as histograms. Vertical dotted red lines indicate the mean cell length. The number of MipZ-CFP foci (red) per cell was also quantified and plotted as histograms.

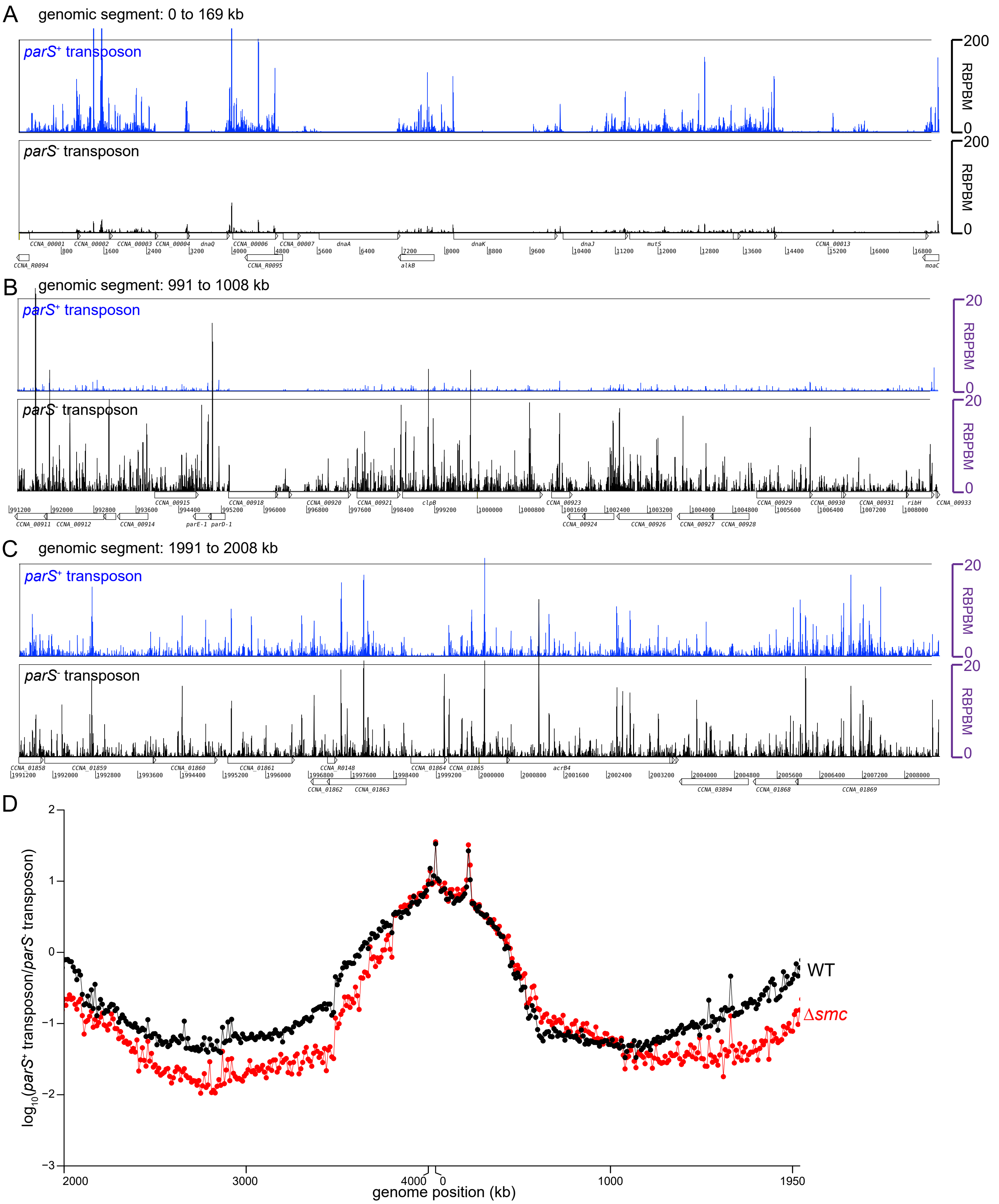

Figure S8. Tn5-seq reveals the positional bias of the centromeric *parS* site on *Caulobacter* chromosome. (A) A comparison of *parS*⁺ (blue) and *parS*⁻ (black) transposon insertions for the genomic segment between +0 kb and +168 kb (*ori* proximal). **(B)** A comparison of *parS*⁺ (blue) and *parS*⁻ (black) transposon insertions for the genomic segment between +9912 kb and +1008 kb (mid-arm). **(C)** A comparison of *parS*⁺ (blue) and *parS*⁻ (black) transposon insertions for the genomic segment between +1991 kb and +2008 kb (*ter* proximal). Axes labeled in purple color highlights different scaling. **(D)** Wild-type (black) or *Δsmc* (red) *Caulobacter* cells were mutagenized with *parS*⁺ or *parS*⁻ transposon, and the number of insertions was binned to 10-kb segments along the *Caulobacter* chromosome. The ratio between insertion frequency for *parS*⁺ transposon and that of *parS*⁻ transposon was calculated and plotted as a $\log_{10}$ scale against genomic position.

MATERIALS AND METHODS

Plasmids and Strains construction

All strains used are listed in Supplementary Table S1. All plasmids and primers used in strain and plasmid construction are listed in Supplementary Table S2.

pMT675::*flag-parB* (WT)

The coding sequence of ParB (CCNA_03868) was amplified from the *Caulobacter* genomic DNA by PCR using primers flag_parB_F_part1 and flag_parB_R. The PCR product was purified using a Qiagen PCR purification column and used as a template in a second PCR (primer: flag_parB_R and flag_parB_part2) to attach the sequence encoding a flexible linker (GGGS) to *parB*. The resulting PCR product was purified and used as a template in the third PCR (primer: flag_parB_R and flag_parB_part3) to attach the sequence of the FLAG tag to *parB*. The final PCR product was gel-purified and assembled to an NdeI-NheI-cut pMT675 (1) using a 2x Gibson master mix (NEB). Briefly, 2.5 µL of each DNA fragment at equimolar concentration was added to 5 µL Gibson master mix (NEB), and the mixture was incubated at 50°C for 60 minutes. 5 µL was used to transform chemically-competent *E. coli* DH5α cells. Gibson assembly was possible due to a 23 bp sequence shared between the PCR fragment and the NdeI-NheI-cut pMT675 backbone. The resulting plasmid was sequence verified by Sanger sequencing (Eurofins, Germany).

pMT675::*flag-parB* (G101S)

To introduce point mutation to the coding sequence of ParB, a pair of primers: parB_G101S_F and parB_G101S_R were used in a PCR to amplify around the pMT675::*parB* (WT) plasmid. DpnI (1 µL) was added to the 50 µL PCR reaction to remove circular template plasmid. The PCR product was precipitated and used to transform chemically-competent *E. coli* DH5α cells. The resulting plasmid was sequenced by Sanger sequencing (Eurofins, Germany) to confirm the successful introduction of the intended mutation.

pMT675::*flag-parB* (R104A)

The same procedure as above was used to mutagenize arginine 104 to alanine, except that primers:parB_R104A_F and parB_R104A_R were used for PCR.

pET21b::ParB-$(His)_6$, pET21b::ParB(G101S)-$(His)_6$, pET21b::ParB(R104A)-$(His)_6$

pET21b-ParB-$(His)_6$ is a gift from Christine Jacobs-Wagner (2). To introduce point mutation to the coding sequence of ParB, a pair of primers: parB_G101S_F and parB_G101S_R (or parB_R104A_F and parB_R104A_R) were used in a PCR to amplify around the pET21b-ParB-$(His)_6$ plasmid. DpnI (1 µL) was added to the 50 µL PCR reaction to remove circular template plasmid. The PCR product was precipitated and used to transform chemically-competent *E. coli* DH5α cells. The resulting plasmid was sequenced by Sanger sequencing (Eurofins, Germany) to confirm the successful introduction of the intended mutation.

pET21b-Spo0J-$(His)_6$

The gene encoding Spo0J was amplified by PCR from *Bacillus subtilis* genomic DNA using primers 21b-spo0J-F and 21b-spo0J-R. The final PCR product was gel-purified and assembled to an NdeI-HindIII-cut pET21b using a 2x Gibson master mix (NEB). Briefly, 2.5 µL of each DNA fragment at equimolar concentration was added to 5 µL Gibson master mix (NEB), and the mixture was incubated at 50°C for 60 minutes. 5 µL was used to transform chemically-competent *E. coli* DH5α cells. Gibson assembly was possible due to a 23 bp sequence shared between the PCR fragment and the NdeI-HindIII-cut pET21b backbone. The resulting plasmid was sequence verified by Sanger sequencing (Eurofins, Germany).

pENTR::*parB*

The coding sequence of ParB was amplified by PCR from *Caulobacter* genomic DNA using primers parB_entr_F and parB_entr_R. The backbone of pENTR plasmid was amplified by PCR using primers pENTR_gibson_backbone_F and pENTR_gibson_backbone_R from the pENTR-D-TOPO cloning kit (Invitrogen). The resulting PCR product was subsequently treated with DpnI to remove the methylated template DNA. The two PCR fragments were each gel-purified and assembled together using a 2xGibson master mix (NEB). Gibson assembly was possible due to 23 bp sequence shared between the two PCR fragments. These 23 bp regions were incorporated during the primer design to amplify *parB*. The resulting plasmid was sequence verified by Sanger sequencing (Eurofins, Germany).

pENTR::*yfp*

The same procedure as above was employed to assemble the YFP coding sequence to the pENTR backbone. The *yfp* gene was amplified by PCR using primers yfp_pentr_F and yfp_pentr_R, and pMT675 (1) as template.

pML477::*flag-yfp*

The *yfp* gene was recombined into a Gateway-compatible destination vector pML477 via LR recombination reaction (Invitrogen). For LR recombination reactions: 1 µL of purified pENTR::*yfp* was incubated with 1 µL of the destination vector pML477, 1 µL of LR Clonase II mastermix, and 2 µL of water in a total volume of 5 µL. The reaction was incubated for an hour at room temperature before being introduced to DH5α *E. coli* cells by heat-shock transformation. Cells were then plated out on LB agar + spectinomycin. Resulting colonies were restruck onto LB agar + spectinomycin and LB agar + kanamycin. Only colonies that survived on LB + spectinomycin plates were subsequently used for culturing and plasmid extraction.

pUTC18::*parB*

parB was recombined into a Gateway-compatible bacterial-two hybrid destination vector pUTC18-DEST (3) via LR recombination reaction (Invitrogen). For LR recombination reactions: 1 µL of purified pENTR::*parB* was incubated with 1 µL of the destination vector pUTC18-DEST, 1 µL of LR Clonase II master mix, and 2 µL of water in a total volume of 5 µL. The reaction was incubated for an hour at room temperature before being introduced to DH5α *E. coli* cells by heat-shock transformation. Cells were plated out on LB agar + carbecicilin. The resulting colonies were restruck onto LB agar + carbenicilin and LB agar + kanamycin. Only colonies that survived on LB agar + carbenicilin were subsequently used for culturing and plasmid extraction.

pKT25::*parB*

parB was amplified by PCR using primers KT25-parB-F and KT25-parB-R, and pMT675::*flag-parB* as template. The PCR product was gel-purified and assembled to a BamHI-EcoRI-cut pKT25 (Euromedex) using a 2x Gibson master mix (NEB). Gibson assembly was possible due to a 23 bp sequence shared between the PCR fragment and the BamHI-EcoRI-cut pKT25. These 23 bp regions were incorporated during the primer design to amplify *parB.* The resulting plasmid was sequence verified by Sanger sequencing (Eurofins, Germany).

pKTN25::*parA*

parA was amplified by PCR from *Caulobacter* genomic DNA using primers KTN25-parA-F and KTN25-parA-R. The PCR product was gel-purified and digested with BamHI and HindIII. The digested PCR product was further purified using a Qiaquick PCR purification column (Qiagen) before being ligated to BamHI-HindIII-cut pKTN25 (Euromdex) using T4 DNA

ligase (NEB). The ligation reaction was composed of 1 µL of BamHI-HindIII-pKTN25, 8 µL of BamHI-HindIII-cut parA PCR product, 1 µL of T4 ligase buffer, and 0.5 µL of T4 ligase enzyme (NEB). The ligation was incubated at room temperature for an hour. 5 µL was used to transform chemically-competent *E. coli* DH5α cells.

pKTN25::*mipZ*

mipZ was amplified by PCR using primer KTN25-mipZ-F and KTN25-mipZ-R, and *Caulobacter* genomic DNA as template. The PCR product was gel-purified and assembled to a BamHI-HindIII-cut pKTN25 (Euromedex) using a 2x Gibson master mix (NEB). Gibson assembly was possible due to a 23 bp sequence shared between the PCR fragment and the BamHI-HndIII-cut pKTN25. These 23 bp regions were incorporated during the primer design to amplify *mipZ.* The resulting plasmid was sequence verified by Sanger sequencing (Eurofins, Germany).

pMT687::*parS* site 1, 2, 3, 4, 5, 6 and 7

To clone *parS* site 1 into pMT678 (1) , we first used primers parS_site1_F and pMT687_cir_R to amplify the backbone of pMT687 by PCR. Since the 24 bp sequence of *parS* site 1 was incorporated in the primer parS_site1_F, the PCR generated a linear DNA fragment that contains both the backbone of pMT687 and *parS* site 1. DpnI (1 µL) was added to the 50 µL PCR reaction to remove circular methylated template plasmid. The PCR product was then gel-purified, and subsequently phosphorylated using T4 PNK enzyme (NEB). The phosphorylated DNA fragment was religated using T4 DNA ligase (NEB) to regenerate a circular plasmid. 5 µL was used to transform chemically-competent *E. coli* DH5α cells. The resulting plasmid was sequenced by Sanger sequencing (Eurofins, Germany) to confirm the incorporation of *parS* site on pMT687 plasmid.

To clone *parS* site 2 into pMT687, we employed the same procedure as above but using primers parS_site2_F and pMT687_cir_R instead.

To clone *parS* site 3 into pMT687, we employed the same procedure as above but using primers parS_site3_F and pMT687_cir_R instead.

To clone *parS* site 4 into pMT687, we employed the same procedure as above but using primers parS_site4_F and pMT687_cir_R instead.

To clone *parS* site 5 into pMT687, we employed the same procedure as above but using primers parS_site5_F and pMT687_cir_R instead.

To clone *parS* site 6 into pMT687, we employed the same procedure as above but using primers parS_site6_F and pMT687_cir_R instead.

To clone *parS* site 7 into pMT687, we employed the same procedure as above but using primers parS_site7_F and pMT687_cir_R instead.

To clone a scrambled *parS* site 3 into pMT687, we employed the same procedure as above but using primers parS_scrambled_site3_F and pMT687_cir_R instead.

pMCS5-$parS^{3+4}$ at +200kb, +1000 kb and +1800kb

For insertion of a 260 bp sequence containing *Caulobacter parS* sites 3 and 4 at +200 kb on the *Caulobacter* genome, primers label200-NdeI-F and label200-SacI-R were used to amplified a ~500 bp fragment by PCR from the *Caulobacter* genomic DNA. This fragment was 5' phosphorylated by T4 PNK (NEB) before being blunt-end ligated to a SmaI-cut pUC19 (Fermentas). The resulting construct was sequence verified by Sanger sequencing (Eurofins, Germany). The NdeI-SacI-ended insert was then liberated from the pUC19-based plasmid by NdeI and SacI double digestion before being cloned into the same sites on pMCS5::$parS^{3+4}$ (Tran et al., 2017). The construction of pMCS5::$parS^{3+4}$ at +1000 kb were

carried out essentially as above, except the pair of primers used were: label1000-NdeI-F and label1000-SacI-R. The construction of pMCS5::*parS*$^{3+4}$ at +1800 kb was reported previously in Tran et al (2017) (6).

pMCS1-Tn5-ME-R6Kγ-kan^{R}-ME

The transposon delivery plasmid was constructed by Gibson assembling three PCR products (fragment 1 to 3) together. To generate fragment 1: the backbone of pMCS1 (1) was amplified by PCR using primers ampMCS1-F, ampMCS1-R, and pMCS1 as template. The resulting PCR product was treated with 1 µL of DpnI enzyme (NEB) at 37°C for an hour to remove circular methylated template DNA. The PCR product was further purified by gel extraction. To generate fragment 2: the Tn5 transposase-encoding gene together with its promoter was amplified by PCR using primers amp_Tn5_F, amp_Tn5_R, and pIT2 (a gift from Colin Manoil) as template. The resulting PCR product was purified by gel extraction. To generate fragment 3: the transposon cassette (ME-R6Kγ origin-kanamycin^{R}-ME) was amplified by PCR using primers amp_jumpF, amp_jumpR, and Ez-Tn5 (EpiCentre) as template. The resulting PCR product was purified by gel extraction. To assemble three fragments together, 1.7 µL of each fragment (1-3) at equimolar concentration was added to 5 µL Gibson master mix (NEB), and the mixture was incubated at 50°C for 60 minutes. 5 µL was used to transform chemically-competent *E. coli* DH5α cells. Gibson assembly was possible due to 23 bp sequence shared among PCR fragments. These 23 bp regions were incorporated during the primer design. The resulting plasmid was sequence verified by Sanger sequencing (Eurofins, Germany).

pMCS1-Tn5-ME-R6Kγ-kan^{R}-*parS*$^{3+4+5}$-ME

To insert 660 bp sequence containing *parS* sites 3, 4, and 5 into the Tn5 transposon cassette, we first amplify around the pMCS1-Tn5-ME-R6Kγ-kan^{R}-ME plasmid by PCR using primers amp_cirF and amp_cirR. At the end of the PCR, 1 µL of DpnI enzyme (NEB) was added, and the reaction was incubated at 37°C for an hour to remove circular methylated template DNA. Subsequently, the PCR product was purified by gel extraction. To generate DNA fragment containing *parS* sites 3, 4, and 5, PCR was used to amplify a 660 bp fragment from the *Caulobacter* genomic DNA using primers amp_parS_3sites_F and amp_parS_3sites_R. The resulting PCR product was purified by gel extraction. The two DNA fragments were assembled together by Gibson assembly. 2.5 µL of each fragment at equimolar concentration was added to 5 µL Gibson master mix (NEB), and the mixture was incubated at 50°C for 60 minutes. 5 µL was used to transform chemically-competent *E. coli* DH5α cells. Gibson assembly was possible due to 23 bp sequence shared among PCR fragments. These 23 bp regions were incorporated during the primer design. The resulting plasmid was sequence verified by Sanger sequencing (Eurofins, Germany).

Strain TLS1628

Electro-competent *Caulobacter* cells were electroporated with pMT675::*flag-parB* (WT) plasmid to allow for a single integration at the *vanA* locus. The correct integration was subsequently verified by PCR.

Strain TLS1629

Same procedure as above, except that plasmid pMT675::*flag-parB* (G101S) was used.

Strain TLS1630

Same procedure as above, except that plasmid pMT675::*flag-parB* (R104A) was used.

Strain TLS1631
van::P_{van}-1x*flag-parB* (WT), marked with chloramphenicol^{R}, was transduced by phage ΦCr30 from TLS1628 to MT148 to result in TLS1631.

Strain TLS1632
van::P$_{van}$-1xflag-parB (G101S), marked with chloramphenicol^{R}, was transduced by phage ΦCr30 from TLS1629 to MT148 to result in TLS1632.

Strain TLS1633
van::P$_{van}$-1xflag-parB (R104A), marked with chloramphenicol^{R}, was transduced by phage ΦCr30 from TLS1630 to MT148 to result in TLS1633.

Strain TLS1634

Electro-competent *Caulobacter* cells were electroporated with pML477::*flag-yfp* and plated out on PYE + spectinomycin. Resulting colonies were re-struck out on PYE + spectinomycin twice to purify the strain.

Strains TLS1635, TLS1636, and TLS1619

Electro-competent *Caulobacter* cells were electroporated with plasmids pMCS5::*parS^{3+4}* at +200 kb, pMCS5::*parS^{3+4}* at +1000 kb, or pMCS5::*parS^{3+4}* at +1800 kb to allow for a single integration at the site of interest. The correct integration was verified by PCR using a primer specific to the *parS*$^{3+4}$ and another primer upstream of the ~500 bp homologous region used to drive integration.

Strains TLS1764, TLS1765, and TLS1766

200kb::*parS^{3+4}*, marked with tetracycline^{R}, was transduced by phage ΦCr30 from TLS1635 to MT190 to result in TLS1764.

1000kb::*parS^{3+4}*, marked with tetracycline^{R}, was transduced by phage ΦCr30 from TLS1636 to MT190 to result in TLS1765.

1800kb::*parS^{3+4}*, marked with tetracycline^{R}, was transduced by phage ΦCr30 from TLS1637 to MT190 to result in TLS1766.

Strains TLS1768, TLS1769, and TLS1770

mipZ::mipZ-cfp, marked with kanamycin^{R}, was transduced by phage ΦCr30 from ML2397 to TLS1634, TLS1635, and TLS1636 to result in TLS1768, TLS1769, and TLS1770, respectively.

Strains TLS1637 to TLS1650

We use Lambda Red (4) to insert a cassette consisting of 24-bp *Caulobacter parS* site and an apramycin antibiotic resistance gene *aac(3)IV* at the *ybbD* locus on the *E. coli* chromosome. An apramycin resistance cassette was first amplified by PCR using primer apramycinR_F and apramycinR_R, and pSET152 (a gift from Lucy Fouston) as template. The resulting PCR product was purified by gel extraction and further used as a template in a second round of PCR to attach a 24 bp *parS* site to the beginning of the apramycin resistance cassette. Pairs of primers that were used are: ybbD_apramycinR_R and ybbD_parS_site1_F (to attach *parS* site 1), ybbD_apramycinR_R and ybbD_parS_site2_F (to attach *parS* site 2), ybbD_apramycinR_R and ybbD_parS_site3_F (to attach *parS* site 3), ybbD_apramycinR_R and ybbD_parS_site4_F (to attach *parS* site 4), ybbD_apramycinR_R and ybbD_parS_site5_F (to attach *parS* site 5), ybbD_apramycinR_R and ybbD_parS_site6_F (to attach *parS* site 6), ybbD_apramycinR_R and ybbD_parS_site7_F (to attach *parS* site 7), and ybbD_apramycinR_R and ybbD_parS_scrambled_site3_F (to attach a scrambled sequence of *parS* site 3). These forward and reverse primers also carry 49 bp homology to the left or the right of the insertion point at the *ybbD* locus. The resulting PCR products were gel-extracted and electroporated into an arabinose-induced *E. coli* AB1157/pKD46 cells. Colonies that formed on LB + apramycin was restruck on LB +

apramycin and incubated at 42°C to cure of pKD46 plasmid. Finally, the correct insertion of the *parS*-apramycin^R cassette was verified by PCR and Sanger sequencing.

These *ybbD*::*parS*-apramycin^R *E. coli* strains were made competent chemically and transformed with pUTC18::*parB* (WT) or pUTC18::*parB* (G101S) to give TLS1638-TLS1649

Construction of Tn5-seq libraries for Illumina deep sequencing

Illumina deep sequencing libraries were constructed as follows:

1) End repair: the following components were mixed together in a DNA LoBind Eppi tube (Eppendorf): 50 µL of sheared genomic DNA, 10 µL of 10xT4 DNA ligase buffer, 2.5 µL 10mM dNTPs, 28.75 µL of autoclaved MiliQ water, 4 µL of T4 DNA polymerase (3,000 U/mL, NEB), 0.75 µL of Large Klenow Fragment (5,000 U/mL, NEB), and 4 µL of T4 polynucleotide kinase (10,000 U/mL, NEB). The reaction was incubated at room temperature for 30 minutes. End-repaired DNA was then purified by a MinElute Reaction Clean up kit (Qiagen) and eluted out using 30 µL of water.

2) A tailing: the following components were mixed together in a DNA LoBind Eppi tube (Eppendorf): 30 µL of purified DNA from step 1, 4 µL of 10xNEB buffer 2, 4 µL of 2mM dATP, and 3 µL of 3'-5' exo$^-$ Klenow fragment (5,000 U/mL, NEB). The reaction was incubated at 37°C for 40 minutes. DNA was then purified by a MinElute Reaction Clean up kit (Qiagen) and eluted out using 15 µL of water.

3) Adaptor ligation: before this step an adaptor was prepared by annealing two single-stranded DNA oligos together. The sequences of the customised oligos are listed in the Supplementary Table S2. For ligating an A-tailed DNA to the adaptor, the following components was mixed in DNA LoBind Eppi tubes: 15 µL of A-tailed DNA from the previous step, 5 µL of adaptor, 2.5 µL of 10xT4 ligase buffer, 1 µL of water, and 1.5 µL of T4 DNA ligase enzyme. The reaction was incubated at room temperature for 30 minutes. DNA was subsequently purified using a Qiagen MinElute kit and eluted out using 50 µL of water.

4) PCR enrichment for Tn5-insertion junctions: The following components for PCR enrichment was added together: 36.5 µL of water, 0.5 µL of P5-ME primer (stock concentration of 100 µM), Primer 2 (100 µM): 0.5 µL of index primer (PE-index 2 or 4, 5, 6, 7, and 12, each with a different barcode for each separated sample), 1 µL of 10 mM dNTP, 10 µL of 5xHF Phusion buffer, 1.5 µL of 100% DMSO, 5 µL of purified DNA from step 3, and 0.5 µL of Phusion enzyme (NEB). The PCR was performed using the following programme: 98°C for 30 s, 98°C for 10 s, 59°C for 20 s, 72°C for 25 s, repeating for 20 cycles, 72°C for 5 min, and 15°C for 1 minute.

5) DNA purification: DNA was loaded on a 2% agarose gel and a DNA band of desired fragment length was purified from primer dimer DNA and unincorporated oligos. Samples were submitted to the next-generation sequencing facility at the Tufts University. A custom read primer and a custom index read primer were used on a Hiseq2500 to read out the Tn5 insertion junctions and samples' indexes. The sequence of these custom primers are listed in the Supplementary Table S2.

For the list of Tn5-seq libraries in this study, see Supplementary Table S3.

ParB-(His)$_6$ protein purification

Plasmid pET21b-ParB (HexaHistidine tag at the C-terminus of ParB, a gift from Christine Jacob-Wagner) was transformed into *E. coli* BL21/pRARE, and 10 mL overnight culture was used to inoculate 2 L LB medium + carbenicilin + chloramphenicol. Cells were grown at 37°C to OD_{600} of ~0.4. The culture was then cooled to 30°C before isopropyl-β-D-thiogalactopyranoside (IPTG) was added to a final concentration of 0.5 mM. The culture was

left shaking for an additional 3 hrs at 30°C before cells were harvested by centrifugation. Pelleted cells were resuspended in a buffer containing 100 mM Tris-HCl pH 8.0, 300 mM NaCl, 5% (v/v) glycerol, EDTA-free protease inhibitor tablet (Roche), and lysed by sonication (three cycles of 20 s with 40 s resting on ice in between each cycle). The cell debris was removed by centrifugation at 84,000 g for 30 min and the supernatant was filtered through a 0.45 µm membrane before being applied to a 1-ml Ni-loaded Hi-Trap Chelating HP column (GE Healthcare) that had been equilibrated with buffer A [100 mM Tris-HCl, pH 8.0, 300 mM NaCl, 10 mM imidazole]. Protein was eluted from the column using an increasing (10 mM to 500 mM) imidazole gradient in the same buffer. ParB-$(His)_6$ fractions were identified using SDS-PAGE, pooled together, and applied to a Heparin HP column (GE Healthcare) that had been equilibrated with buffer A [100mM Tris-HCl pH 8.0, 25mM NaCl, 5% (v/v) glycerol]. Protein was eluted from the column using an increasing (25 mM to 1 M NaCl) salt gradient in the same buffer A. ParB-$(His)_6$ fractions were identified using SDS-PAGE, pooled together, and concentrated to approximately 2 mg/mL using a Vivaspin6 10 kDa cut-off protein concentrator (Vivascience). The concentrated protein was then exchanged into a storage buffer [50mM Tris-HCl, pH 8.0, 250 mM NaCl and 10% glycerol] using a Zeba desalting column (Thermo Scientific) before flash-frozen in liquid nitrogen. The concentration of ParB dimer was measured by Bradford method before Surface Plasmon Resonance (SPR) experiments. ParB-$(His)_6$ is ~95% pure as judged by SDS-PAGE. ParB mutants: G101S and R104A were purified using the same procedure as ParB (WT) but from 8 L and 6 L of cultures, respectively. *B. subtilis* Spo0J/ParB, expressed from pET21b-Spo0J plasmid, was purified exactly as for *Caulobacter* ParB-$(His)_6$ but from 4 L of cultures.

Bi-parental *E. coli-Caulobacter* conjugation and *parS*$^+$ plasmid toxicity assay

E. coli S17-1 cells were transformed with low-copy number plasmids containing individual *parS* site by heat shock and plated out on LB + kanamycin. Resulting colonies were grown to mid-exponential phase in preparation for a bi-parental conjugation with the wild-type *Caulobacter* CB15N. Briefly, *E. coli* cells were pelleted and resuspended in fresh PYE to wash off residual antibiotics from the growing culture. 100 µL of *E. coli* cells at OD_{600} of 0.4 was mixed with 500 µL of exponentially growing *Caulobacter* at OD_{600} of 0.4. The mixture of *E. coli* and *Caulobacter* cells were centrifuged at 13,000 rpm for 1 minute and the pellet was resuspended in 50 µL of fresh PYE before being spotted on a nitrocellulose membrane. The membrane was then laid on top of a fresh PYE plate and incubated at 30°C for 5 hrs. After the incubation, cells were released from the nitrocellulose membrane by vortexing in an Eppendorf tube containing 500 µL of fresh PYE. A ten-fold serial dilution was performed for each conjugation and 5 µL of each dilution was spotted on PYE plates supplemented with just nalidixic acid or with both nalidixic acid and kanamycin. Plates were incubated at 30°C for 3 days to allow *Caulobacter* colonies to form.

Immunoblot analysis

For Western blot analysis, *Caulobacter* or *E. coli* cells were pelleted and resuspended directly in 1xSDS sample buffer, then heated to 95°C for 5 min before loading. Total protein was run on 10% Tris-HCl gels (Bio-Rad) at 150 V for separation. Resolved proteins were transferred to polyvinylidene fluoride membranes using the Trans-Blot Turbo Transfer System (BioRad) and probed with 1:10,000 dilution of primary α-FLAG antibodies (Sigma-Aldrich), or 1:5,000 dilution of α-T18 antibody (Abcam) and subsequently by a secondary HRP–conjugated antibody (1:5,000). Blots were imaged using an Amersham Imager 600 (GE Healthcare), and quantified using ImageStudio Lite (Licor).

Bacterial-two hybrid assay

Bacterial-two hybrid assays were performed exactly as described in the Euromedex manual. Briefly, *E. coli* BTH101 cells were co-transformed with a pair of plasmids by electroporation before being plated out on LB + carbenicilin + kanamycin and incubated at 30°C. Three

colonies from each plasmid combination were grown up in LB + carbenicilin + kanamycin overnight. 5 µL of overnight cultures were spotted on McConkey plates supplemented with carbenicilin, kanamycin, 1% maltose and 1mM IPTG. Plates were incubated at 30°C and their picture were taken after 2 days. To measure β-galactosidase activities, we also used overnight cultures. Three biological replicates were performed to obtain the mean and standard deviation of β-galactosidase activities.

TABLE S1: Strains

**Strains**	**Description**	**Source**
Caulobacter crescentus		
CB15N	Wild-type synchronizable *Caulobacter crescentus*	Lab collection
MT148	CB15N *parB*::P$_{xyl}$-*parB mipZ::mipZ-yfp*	gift from Martin Thanbichler (5)
MT190	CB15N *parB::cfp-parB*	gift from Martin Thanbichler (5)
TLS 1628	CB15N *van::P$_{van}$*-1x*flag-parB* (WT)	This study
TLS 1629	CB15N *van::P$_{van}$*-1x*flag-parB* (G101S)	This study
TLS 1630	CB15N *van::P$_{van}$*-1x*flag-parB* (R104A)	This study
TLS 1631	CB15N *parB*::P$_{xyl}$-*parB van::P$_{van}$*-1x*flag-parB* (WT)	This study
TLS 1632	CB15N *parB*::P$_{xyl}$-*parB van::P$_{van}$*-1x*flag-parB* (G101S)	This study
TLS 1633	CB15N *parB*::P$_{xyl}$-*parB van::P$_{van}$*-1x*flag-parB* (R104A)	This study
TLS 1634	CB15N pML477::1x*flag-yfp*	This study
TLS 1635	CB15N 200 kb::*parS^{3+4}*	This study
TLS 1636	CB15N 1000 kb::*parS^{3+4}*	This study
TLS 1619	CB15N 1800 kb::*parS^{3+4}*	Le lab collection (6)
TLS 1764	CB15N *parB::cfp-parB* 200 kb::*parS^{3+4}*	This study
TLS 1765	CB15N *parB::cfp-parB* 1000 kb::*parS^{3+4}*	This study
TLS 1766	CB15N *parB::cfp-parB* 1800 kb::*parS^{3+4}*	This study
ML 2397	CB15N *mipZ::mipZ-cfp*	Laub lab collection, gift from A. Badrinarayanan
TLS 1768	CB15N *mipZ::mipZ-cfp* 200 kb::*parS^{3+4}*	This study
TLS 1769	CB15N *mipZ::mipZ-cfp* 1000 kb::*parS^{3+4}*	This study
TLS 1770	CB15N *mipZ::mipZ-cfp* 1800 kb::*parS^{3+4}*	This study
Escherichia coli		
AB1157	*thr-1, ara-14, leuB6, Δ(gpt-proA)62, lacY1, tsx-33, supE44, galK2, rac-, hisG4(Oc), rfbD1, mgl-51, rpsL31, kdgK51, xyl-5, mtl-1, argE3 (Oc), thi-1, qsr-*	Yale *E. coli* Genetic Stock Center
DH5α	*E. coli* host for DNA cloning and propagation of plasmid	Le lab collection
BTH101	*cya- E. coli* host for bacterial-two hybrid assay	Euromedex
S17-1	*E. coli* host for *E. coli-Caulobacter* conjugative plasmids	Le lab collection
BL21	*E. coli* host for protein overexpression from an IPTG-inducible T7 promoter	Le lab collection

CJW4025	BL21 pET21b::*parB-(his)6*	gift from Christine Jacob-Wagner (2)
TLS1665	BL21 pET21b::*parB(G101S)-(his)6*	This study
TLS1666	BL21 pET21b::*parB(R104A)-(his)6*	This study
TLS 1637	AB1157 *ybbD::parS* site 1 pUTC18::*parB* (WT)	This study
TLS 1638	AB1157 *ybbD::parS* site 2 pUTC18::*parB* (WT)	This study
TLS 1639	AB1157 *ybbD::parS* site 3 pUTC18::*parB* (WT)	This study
TLS 1640	AB1157 *ybbD::parS* site 4 pUTC18::*parB* (WT)	This study
TLS 1641	AB1157 *ybbD::parS* site 5 pUTC18::*parB* (WT)	This study
TLS 1642	AB1157 *ybbD::parS* site 6 pUTC18::*parB* (WT)	This study
TLS 1643	AB1157 *ybbD::parS* site 7 pUTC18::*parB* (WT)	This study
TLS 1644	AB1157 *ybbD::parS* scrambled site 3 pUTC18::*parB* (WT)	This study
TLS 1645	AB1157 *ybbD::parS* site 2 pUTC18::*parB* (G101S)	This study
TLS 1646	AB1157 *ybbD::parS* site 3 pUTC18::*parB* (G101S)	This study
TLS 1647	AB1157 *ybbD::parS* site 4 pUTC18::*parB* (G101S)	This study
TLS 1648	AB1157 *ybbD::parS* site 5 pUTC18::*parB* (G101S)	This study
TLS 1649	AB1157 *ybbD::parS* site 7 pUTC18::*parB* (G101S)	This study
TLS 1650	AB1157 *ybbD::parS* scrambled site 3 pUTC18::*parB* (G101S)	This study
TLS 1651	BTH101 pUTC18::*parB* pKT25::*parB*	This study
TLS 1652	BTH101 pUTC18::*parB* pKTN25::*parA*	This study
TLS 1653	BTH101 pUTC18::*parB* pKT25::*mipZ*	This study
TLS 1654	BTH101 pUTC18::*parB* pKT25 empty	This study
TLS 1655	BTH101 pUTC18::*zip* pKT25::*zip*	This study
TLS 1656	S17-1 pMT687::*parS* site 1	This study
TLS 1657	S17-1 pMT687::*parS* site 2	This study
TLS 1658	S17-1 pMT687::*parS* site 3	This study
TLS 1659	S17-1 pMT687::*parS* site 4	This study
TLS 1660	S17-1 pMT687::*parS* site 5	This study
TLS 1661	S17-1 pMT687::*parS* site 6	This study
TLS 1662	S17-1 pMT687::*parS* site 7	This study
TLS 1663	S17-1 pMCS1-Tn5-ME-R6Kγ-kan^R-ME	This study
TLS 1664	S17-1 pMCS1-Tn5-ME-R6Kγ-kan^R-*par*$^{3+4+5}$-ME	This study
TLS 1667	BL21 pET21b::spo0J-*(his)6*	This study

TABLE S2. PLASMIDS AND PRIMERS

**Plasmids**	**Description**	**Source**
pENTR-D-TOPO	ENTRY vector for gateway cloning, kanamycin^R	Invitrogen
pMT675	integrative vector to the *vanA* locus, chloramphenicol^R	(1)
pMCS1	non-replicative, suicide vector in *Caulobacter*, pMB1 origin of replication, spectinomycin^R	(1)
pUTC18-DEST	Gateway-cloning destination vector for bacterial-two hybrid assay, carbenicilin^R	Gift from Daniel Ladant

		(3)
pKT25	vector for bacterial-two hybrid assay, kanamycin^R	Euromedex
pKTN25	vector for bacterial-two hybrid assay, kanamycin^R	Euromedex
pML477	Gateway-cloning destination vector for fusion of protein interest to an N-terminally FLAG tag, xylose-inducible promoter, high-copy number plasmid, spectinomycin^R	Laub lab strain collection
pENTR::*parB*	entry vector harboring the coding sequence of ParB, kanamycin^R	This study
pENTR::*yfp*	entry vector harboring the coding sequence of YFP, kanamycin^R	This study
pML477::*flag-yfp*	high-copy number plasmid expressing *flag-yfp*, spectinomycin^R	This study
pMT675::*flag-parB*	integrative vector, for expression of *flag-parB* from the *vanA* locus, chloramphenicol^R	This study
pMT675::*flag-parB* (G101S)	integrative vector, for expression of *flag-parB* (G101S) from the *vanA* locus, chloramphenicol^R	This study
pMT675::*flag-parB* (R104A)	integrative vector, for expression of *flag-parB* (R104A) from the *vanA* locus, chloramphenicol^R	This study
pMT678::*parS* site 1	replicative low-copy number plasmid carrying 24 bp *parS* site number 1, carbenicilin^R and kanamycin^R	This study
pMT678::*parS* site 2	replicative low-copy number plasmid carrying 24 bp *parS* site number 2, carbenicilin^R and kanamycin^R	This study
pMT678::*parS* site 3	replicative low-copy number plasmid carrying 24 bp *parS* site number 3, carbenicilin^R and kanamycin^R	This study
pMT678::*parS* site 4	replicative low-copy number plasmid carrying 24 bp *parS* site number 4, carbenicilin^R and kanamycin^R	This study
pMT678::*parS* site 5	replicative low-copy number plasmid carrying 24 bp *parS* site number 5, carbenicilin^R and kanamycin^R	This study
pMT678::*parS* site 6	replicative low-copy number plasmid carrying 24 bp *parS* site number 6, carbenicilin^R and kanamycin^R	This study
pMT678::*parS* site 7	replicative low-copy number plasmid carrying 24 bp *parS* site number 7, carbenicilin^R and kanamycin^R	This study
pMT678::*parS* scrambled site 3	replicative low-copy number plasmid carrying a scrambled 24 bp *parS* site 3, carbenicilin^R and kanamycin^R	This study
pMCS5::*parS*$^{3+4}$	plasmid for insertion of *parS* sites 3 and 4 at an ectopic location on the *Caulobacter* chromosome, tetracycline^R	Le lab collection, (6)
pMCS5::*parS*$^{3+4}$ at +200 kb	label +200 kb with *Caulobacter parS* site 3 and 4, tetracycline^R	This study
pMCS5::*parS*$^{3+4}$ at +1000 kb	label +1000 kb with *Caulobacter parS* site 3 and 4, tetracycline^R	This study
pMCS5::*parS*$^{3+4}$ at +1800 kb	label +1800 kb with *Caulobacter parS* site 3 and 4, tetracycline^R	Le lab collection, (6)
pET21b-ParB-(His)$_6$	overexpression of ParB-(His)$_6$ from an IPTG-inducible T7 promoter	gift from Christine Jacob-Wagner (2)
pET21b-Spo0J-(His)$_6$	overexpression of Spo0J-(His)$_6$ from an IPTG-inducible T7 promoter	This study

pET21b-ParB(G101S)-(His)$_6$	overexpression of ParB (G101S)-(His)$_6$ from an IPTG-inducible T7 promoter	This study
pET21b-ParB(R104A)-(His)$_6$	overexpression of ParB (R104A)-(His)$_6$ from an IPTG-inducible T7 promoter	This study
pUTC18::*parB*	bacterial-two hybrid vector expressing T18-ParB (WT), carbenicilin^R	This study
pUTC18::*parB* (G101S)	bacterial-two hybrid vector expressing T18-ParB (G101S), carbenicilin^R	This study
pUTC18::*parB* (R104A)	bacterial-two hybrid vector expressing T18-ParB (R104A), carbenicilin^R	This study
pKTN25-*parA*	bacterial-two hybrid vector expressing ParA-T25, kanamycin^R	This study
pKT25::*mipZ*	bacterial-two hybrid vector expressing T25-MipZ, kanamycin^R	This study
pKT25::*parB*	bacterial-two hybrid vector expressing T25-ParB, kanamycin^R	This study
pUTC18::*zip*	bacterial-two hybrid vector expressing T18-ZIP, carbenicilin^R	Euromedex
pKT25::*zip*	bacterial-two hybrid vector expressing T25-ZIP, kanamycin^R	Euromedex
pMCS1-Tn5-ME-R6Kγ-kan^R-ME	Tn5 transposon delivery containing Tn5 transposase-encoding gene and a ME-R6Kγ-kanamycin^R-ME cassette, kanamycin^R and spectinomycin^R	This study
pMCS1-Tn5-ME-R6Kγ-kan^R-*parS*$^{3+4+5}$-ME	Tn5 transposon delivery containing Tn5 transposase-encoding gene and a ME-R6Kγ-*parS*$^{3+4+5}$-kanamycin^R-ME cassette, kanamycin^R and spectinomycin^R	This study

**Oligonucleotides (Oligos)**	**Sequence**
**Oligos for construction of pMT675::*flag-parB***	
flag-parB_R	CTAGTGGATCCCCCGGGCTGCAGCTAGCTCAGATCCCGCGCGTCAGTCG
flag-parB_part1	GGCGGCGGCTCGGGCGGCGGCGGCTCGATGGAGTCCGTCGTGGTGGGAG
flag-parB_part2	AAGGACGACGACGACAAGGGCGGCGGCGGCTCGGGCGGCGGCGGCTCG
flag-parB_part3	CCGAACCACGATGCGAGGAAACGCATATGGACTACAAGGACGACGACGA
**Site-directed mutagenesis oligos to generate *parB* (G101S)**	
parB_G101S_F	CCAGATCGTCGCCAGCGAGCGCCGTTG
parB_G101S_R	CAACGGCGCTCGCTGGCGACGATCTGG

**Site-directed mutagenesis oligos to generate *parB* (R104A)**	
parB_R104A_F	CGCCGGCGAGCGCGCCTGGCGCGCAGCCC
parB_R104A_R	GGGCTGCGCGCCAGGCGCGCTCGCCGGCG
**For the construction of pENTR::*parB***	
parB_entr-F	GCTCCGCGGCCGCCCCCTTCACCATGGAGTCCGTCGTGGTGGGAGAGCC
parB_entr-R	GCTGGGTCGGCGCGCCCACCCTTTCAGATCCCGCGCGTCAGTCGGTTGC
pENTR_gibson_backbone_R	GGTGAAGGGGGCGGCCGCGGAGCCTGC
pENTR_gibson_backbone_F	AAGGGTGGGCGCGCCGACCCAGCTTTCTTG
**For the construction of pENTR::*yfp***	
yfp_entr-F	CTCCGCGGCCGCCCCCTTCACCATGGTGAGCAAGGGCGAGGAGCTGTTC
yfp_entr-R	CTGGGTCGGCGCGCCCACCCTTTTACTTGTACAGCTCGTCCATGCCGAG
**For the construction of pKT25::*parB***	
KT25-parB-F	CGGGCTGCAGGGTCGACTCTAGAGGATCCCGGTGGTGGTTCCATGTCCGAAGGG
KT25-parB-R	AGTCACGACGTTGTAAAACGACGGCCGAATTCTCAGATCCCGCGCGTCAGTCGG
**For the construction of pKTN25::*parA***	
KTN25-parA-F	CAGCTATGACCATGATTACGCCAAGCTTGGTGTCCGCTAATCCTCTCCGCG
KTN25-parA-R	CTGAATTCGAGCTCGGTACCCGGGGATCCTCGGCGGCCTTGGCCTGGCGATCG
**For the construction of pKTN25::*mipZ***	
KTN25-mipZ-F	TATGACCATGATTACGCCAAGCATGGCCGAAACGCGCGTTATCGTCGTC
KTN25-mipZ-R	TGAATTCGAGCTCGGTACCCGGGGCTGCGCCGCCAGCATCGTCTCGCCG
**Oligos for construction of pMT687 ::*parS***	
pMT687_cir_R	ATGGCGAATGGCGCCGCGCTGATGTCCGGCGGTG
parS_site1_F	ATGCCAAGCCCCGTGGAACAGTTGATGGCGCCTGATGCGGTATTTTCTCCTTAC
parS_site2_F	CGGTGTTCCACGTGAAAACCTCAGATGGCGCCTGATGCGGTATTTTCTCCTTAC
parS_site3_F	TCGGCGTTTCACGTGAAACACCCCATGGCGCCTGATGCGGTATTTTCTCCTTAC

parS_site4_F	GGGATGTTCCACGTGAAACATCACATGGCGCCTGATGCGG TATTTTCTCCTTAC
parS_site5_F	GCTTGTTTCACGTGAAAACCATCGATGGCGCCTGATGCGGT ATTTTCTCCTTAC
parS_site6_F	CCCGCCGCCCCCGTGAAACGTCCGATGGCGCCTGATGCGG TATTTTCTCCTTAC
parS_site7_F	CGATGTTTCCACGTGAAACAAGGCATGGCGCCTGATGCGG TATTTTCTCCTTAC
parS_scrambled_site4_ F	GGCTACACGACGTCTCGCTCACTAATGGCGCCTGATGCGG TATTTTCTCCTTAC
**For construction of pMCS5::*parS*$^{3+4}$ at +200 kb**	
label200_NdeI_F	CATATGATCGAAAAGACCTTCAAGCTG
label200_SacI_R	GAGCTCTCACGCCTTTCCCATATAGATGAAC
**For construction of pMCS5::*parS*$^{3+4}$ at +1000 kb**	
label1000_NdeI_F	CATATGTTGGGCTTAGGTGTGGACCACG
label1000_SacI_R	GAGCTCCTACCGCCGCTTCAACTTCGCCAG
**Oligos for construction of Tn5 delivery plasmid**	
ampMCS1-R	CCCTGCTTCGGGGTCATTATAGCG
ampMCS1-F	TGCAGCCCGGGGGATCCACTAGTTCTAGAGCGGC
ampTn5F	CGCTATAATGACCCCGAAGCAGGGGTCGACCGATCCCGTA CACAAGTAG
ampTn5R	TCAGATCTTGATCCCCTGCGCCATCAGATC
amp_jumpF	TGGCGCAGGGGATCAAGATCTGACTGTCTCTTATACACATC TCAACCATCATCGATGAATTGC
amp_jumpR	CTAGTGGATCCCCCGGGCTGCACTGTCTCTTATACACATCT CAACCCTGAAGCTTGCATGCCTG
amp_cirF	GGGATCTGCCATTTCATTACCTCTTTCTCCGC
amp_cirR	TGGCTTGTTGTCCACAACCGTTAAACCTTAAAAGCTTTAAAA G
amp_parS_3sites_F	TAACGGTTGTGGACAACAAGCCAGTTCGAGCGGGGGCGCT GGACTCGAT
amp_parS_3sites_R	GAGGTAATGAAATGGCAGATCCCGATGTGATCGAGGCCGA GGGTGTTCG
**Oligos for construction of strain with individual *parS* and apramycin^{R} *aac(3)IV* cassette inserted at the *ybbD* locus**	
apramycinR_F	ATCCTTTTGGTTCATGTGCAGCTCC
apramycinR_R	TCAGCCAATCGACTGGCGAGCGGC
ybbD_apramycinR_R	TTGACGACTTCGATATGGGATAGACTCTTAATTCAAGCAATC AGCCAATCGACTGGCGAGCG
ybbD_parS_site1	AATATTGGAGCTGGATTGCCTGATGCTTGTGCAGAGTAAAT GCCAAGCCCCGTGGAACAGTTGATCCTTTTGGTTCATGTGC

	AGCTC
ybbD_parS_site2	AATATTGGAGCTGGATTGCCTGATGCTTGTGCAGAGTAACGGTGTTCCACGTGAAAACCTCAGATCCTTTTGGTTCATGTGCAGCTC
ybbD_parS_site3	AATATTGGAGCTGGATTGCCTGATGCTTGTGCAGAGTAATCGGCGTTTCACGTGAAACACCCCATCCTTTTGGTTCATGTGCAGCTC
ybbD_parS_site4	AATATTGGAGCTGGATTGCCTGATGCTTGTGCAGAGTAAGGGATGTTCCACGTGAAACATCACATCCTTTTGGTTCATGTGCAGCTC
ybbD_parS_site5	AATATTGGAGCTGGATTGCCTGATGCTTGTGCAGAGTAAGCTTGTTTCACGTGAAAACCATCGATCCTTTTGGTTCATGTGCAGCTC
ybbD_parS_site6	AATATTGGAGCTGGATTGCCTGATGCTTGTGCAGAGTAACCCGCCGCCCCCGTGAAACGTCCGATCCTTTTGGTTCATGTGCAGCTC
ybbD_parS_site7	AATATTGGAGCTGGATTGCCTGATGCTTGTGCAGAGTAACGATGTTTCCACGTGAAACAAGGCATCCTTTTGGTTCATGTGCAGCTC
ybbD_parS_scrambled_site3	AATATTGGAGCTGGATTGCCTGATGCTTGTGCAGAGTAAGGCTACACGACGTCTCGCTCACTAATCCTTTTGGTTCATGTGCAGCTC
**Oligos for construction of pET21b::*spo0J-(His)$_6$***	
21b-spo0J-F	TTAACTTTAAGAAGGAGATATACATATGGCTAAAGGCCTTGGAAAAGGGATTAATGCG
21b-spo0J-R	GGTGGTGCTCGAGTGCGGCCGCAAGCTTTGATTCTCGTTCAGACAAAAGCTCTAAAATC
**Oligos for REDCAT SPR**	
F-redcat-S1	ATGCCAAGCCCCGTGGAACAGTTG
R-redcat-S1	CAACTGTTCCACGGGGCTTGGCATCCTACCCTACGTCCTCCTGC
F-redcat-S2	CGGTGTTCCACGTGAAAACCTCAG
R-redcat-S2	CTGAGGTTTTCACGTGGAACACCGCCTACCCTACGTCCTCCTGC
F-redcat-S3	TCGGCGTTTCACGTGAAACACCCC
R-redcat-S3	GGGGTGTTTCACGTGAAACGCCGACCTACCCTACGTCCTCCTGC
F-redcat-S4	GGGATGTTCCACGTGAAACATCAC
R-redcat-S4	GTGATGTTTCACGTGGAACATCCCCCTACCCTACGTCCTCCTGC
F-redcat-S5	GCTTGTTTCACGTGAAAACCATCG
R-redcat-S5	CGATGGTTTTCACGTGAAACAAGCCCTACCCTACGTCCTCCTGC
F-redcat-S6	CCCGCCGCCCCCGTGAAACGTCCG
R-redcat-S6	CGGACGTTTCACGGGGGCGGCGGGCCTACCCTACGTCCTCCTGC
F-redcat-S7	CGATGTTTCCACGTGAAACAAGGC
R-redcat-S7	GCCTTGTTTCACGTGGAAACATCGCCTACCCTACGTCCTCC

	TGC
F-redcat-Scramble3	GGCTACACGACGTCTCGCTCACTA
R-redcat-Scramble3	TAGTGAGCGAGACGTCGTGTAGCCCCTACCCTACGTCCTCC TGC
Bacillus-F-redcat-S	AGAATGTTCCACGTGAAACAAAGA
Bacillus-R-redcat-S	TCTTTGTTTCACGTGGAACATTCTCCTACCCTACGTCCTCCT GC
Bacillus-F-redcat-Scramble	CAATAATAGACCGTAGTGAGAACA
Bacillus-R-redcat-Scramble	TGTTCTCACTACGGTCTATTATTGCCTACCCTACGTCCTCCT GC
**Oligos for Tn5-seq experiments**	
adaptor_S	p-GATCGGAAGAGCGGTTCAGCAGGAATGCCGAG (p: phosphate group)
adaptor_AS	ACACTCTTTCCCTACACGACGCTCTTCCGATC*T (*:phosphorothioate bond)
P5-ME	AATGATACGGCGACCACCGAGATCTACGGTTGAGATGTGTA TAA GAGACAG
PE-Index2	CAAGCAGAAGACGGCATACGAGAT ACATCG CGGTCTCGGCATTCCTGCTGAACCGCTCTTCCGATC
PE-Index4	CAAGCAGAAGACGGCATACGAGAT TGGTCA CGGTCTCGGCATTCCTGCTGAACCGCTCTTCCGATC
PE-Index5	CAAGCAGAAGACGGCATACGAGAT CACTGT CGGTCTCGGCATTCCTGCTGAACCGCTCTTCCGATC
PE-Index6	CAAGCAGAAGACGGCATACGAGAT ATTGGC CGGTCTCGGCATTCCTGCTGAACCGCTCTTCCGATC
PE-Index7	CAAGCAGAAGACGGCATACGAGAT GATCTG CGGTCTCGGCATTCCTGCTGAACCGCTCTTCCGATC
PE-Index12	CAAGCAGAAGACGGCATACGAGAT TACAAG CGGTCTCGGCATTCCTGCTGAACCGCTCTTCCGATC
sequence_read_primer	ACCGAGATCTACGGTTGAGATGTGTATAAGAGACAG
index_read_primer	GATCGGAAGAGCGGTTCAGCAGGAATGCCGAGACCG

TABLE S3. ChIP-seq and Tn5-seq datasets

**ChIP-seq datasets**	**Fragment length**	**GEO**
CB15N *parB*::P$_{xyl}$-*parB van::P$_{van}$*-1x*flag-parB* (WT), fixation with 1% formadehyde, α-FLAG antibody (Sigma), ChIP fraction	146±56 bp	GSE100233 This study
CB15N *parB*::P$_{xyl}$-*parB van::P$_{van}$*-1x*flag-parB* (G101S), fixation with 1% formadehyde, α-FLAG antibody (Sigma), ChIP fraction	129±53 bp	GSE100233 This study
CB15N *parB*::P$_{xyl}$-*parB van::P$_{van}$*-1x*flag-parB* (R104A), fixation with 1% formadehyde, α-FLAG antibody (Sigma), ChIP fraction	135±56 bp	GSE100233 This study
CB15N pML477::1x*flag-yfp*, fixation with 1% formadehyde, α-FLAG antibody (Sigma), ChIP fraction	123±52 bp	GSE100233 This study
CB15N *parB*::cfp-*parB*, fixation with 1% formadehyde, α-GFP antibody (Abcam), ChIP fraction	164±64 bp	GSE100233 This study
CB15N, fixation with 1% formadehyde, α-ParB	138±45 bp	GSE100233

polyclonal antibody, ChIP fraction		This study
AB1157 *ybbD::parS* site 4 pUTC18::*parB* (WT)	178±55 bp	GSE100233 This study
AB1157 *ybbD::parS* site 3 pUTC18::*parB* (WT)	171±53 bp	GSE100233 This study
AB1157 *ybbD::parS* site 7 pUTC18::*parB* (WT)	168±44 bp	GSE100233 This study
AB1157 *ybbD::parS* site 2 pUTC18::*parB* (WT)	163±51 bp	GSE100233 This study
AB1157 *ybbD::parS* site 5 pUTC18::*parB* (WT)	173±54 bp	GSE100233 This study
AB1157 *ybbD::parS* scrambled site 3 pUTC18::*parB* (WT)	165±47 bp	GSE100233 This study
AB1157 *ybbD::parS* site 6 pUTC18::*parB* (WT)	175±53 bp	GSE100233 This study
AB1157 *ybbD::parS* site 1 pUTC18::*parB* (WT)	168±51 bp	GSE100233 This study
AB1157 *ybbD::parS* site 4 pUTC18::*parB* (G101S)	136±41 bp	GSE100233 This study
AB1157 *ybbD::parS* site 3 pUTC18::*parB* (G101S)	167±59 bp	GSE100233 This study
AB1157 *ybbD::parS* site 7 pUTC18::*parB* (G101S)	136±42 bp	GSE100233 This study
AB1157 *ybbD::parS* site 2 pUTC18::*parB* (G101S)	154±49 bp	GSE100233 This study
AB1157 *ybbD::parS* site 5 pUTC18::*parB* (G101S)	145±49 bp	GSE100233 This study
AB1157 *ybbD::parS* scrambled site 3 pUTC18::*parB* (G101S)	131±41 bp	GSE100233 This study
**IDAP-seq datasets**		
ParB-$(His)_6$ with sonicated *Caulobacter crescentus* genomic DNA	ND	GSE100233 This study
No protein control **Tn5-seq datasets**	ND	GSE100233 This study
CB15N randomly mutagenized with *parS*$^-$ transposon	ND	GSE100233 This study
CB15N randomly mutagenized with *parS*$^+$ transposon	ND	GSE100233 This study
CB15N *Δsmc* randomly mutagenized with *parS*$^-$ transposon	ND	GSE100233 This study
CB15N *Δsmc* randomly mutagenized with *parS*$^+$ transposon	ND	GSE100233 This study
CB15N Flip1-5 randomly mutagenized with *parS*$^-$ transposon	ND	GSE100233 This study
CB15N Flip1-5 randomly mutagenized with *parS*$^+$ transposon	ND	GSE100233 This study
CB15N Flip2-5 randomly mutagenized with *parS*$^-$ transposon	ND	GSE100233 This study
CB15N Flip2-5 randomly mutagenized with *parS*$^+$ transposon	ND	GSE100233 This study

ND: Not determined due to single-end Illumina sequencing

REFERENCES

1. Thanbichler,M., Iniesta,A.A. and Shapiro,L. (2007) A comprehensive set of plasmids for vanillate- and xylose-inducible gene expression in *Caulobacter crescentus*. *Nucleic Acids Res.*, **35**, e137–e137.

2. Lim,H.C., Surovtsev,I.V., Beltran,B.G., Huang,F., Bewersdorf,J. and Jacobs-Wagner,C. (2014) Evidence for a DNA-relay mechanism in ParABS-mediated chromosome segregation. *eLife*, **3**, e02758.

3. Ouellette,S.P., Gauliard,E., Antosová,Z. and Ladant,D. (2014) A Gateway(®) -compatible bacterial adenylate cyclase-based two-hybrid system. *Environ. Microbiol. Rep.*, **6**, 259–267.

4. Datsenko,K.A. and Wanner,B.L. (2000) One-step inactivation of chromosomal genes in *Escherichia coli* K-12 using PCR products. *Proc. Natl. Acad. Sci. U.S.A.*, **97**, 6640–6645.

5. Thanbichler,M. and Shapiro,L. (2006) MipZ, a Spatial Regulator Coordinating Chromosome Segregation with Cell Division in Caulobacter. *Cell*, **126**, 147–162.

6. Tran,N.T., Laub,M.T. and Le,T.B.K. (2017) SMC Progressively Aligns Chromosomal Arms in *Caulobacter crescentus* but Is Antagonized by Convergent Transcription. *Cell Rep.*, **20**, 2057–2071.
